# Is polydopamine beneficial for cells on the modified surface?

**DOI:** 10.1093/rb/rbac078

**Published:** 2022-10-13

**Authors:** Yue Yu, Xiuli Wang, Yi Zhu, Yingning He, Hongrui Xue, Jiandong Ding

**Affiliations:** State Key Laboratory of Molecular Engineering of Polymers, Department of Macromolecular Science, Fudan University, Shanghai 200438, China; State Key Laboratory of Molecular Engineering of Polymers, Department of Macromolecular Science, Fudan University, Shanghai 200438, China; State Key Laboratory of Molecular Engineering of Polymers, Department of Macromolecular Science, Fudan University, Shanghai 200438, China; State Key Laboratory of Molecular Engineering of Polymers, Department of Macromolecular Science, Fudan University, Shanghai 200438, China; State Key Laboratory of Molecular Engineering of Polymers, Department of Macromolecular Science, Fudan University, Shanghai 200438, China; State Key Laboratory of Molecular Engineering of Polymers, Department of Macromolecular Science, Fudan University, Shanghai 200438, China

**Keywords:** biomaterial, polymer, polydopamine, surface modification, cell viability

## Abstract

Since the pioneering work of Messersmith’s group discovering that polydopamine (PDA) can serve to adhere to many types of materials, the PDA coating has, as a biomimetic approach, been widely used to enhance cell adhesion by surface modification to bind biologically active substances to a bioinert substrate. Nevertheless, it is unclear whether or not the PDA itself is beneficial for cells. Herein, we report that a PDA coating decreases viability of cells under normal culture and observation conditions. Such an inhibition effect was not caused by the free PDA or any inherent cytotoxicity of this chemical substance but a contact-dependent phenomenon. Human bone marrow mesenchymal stem cells were employed as the default cell type and tissue culture plates were used as the default substrate, although some other cell types and substrates were also examined to confirm the universality of such an ‘abnormal’ phenomenon of a superstar molecule. The viability of cells on the PDA coating exhibited time dependence, and the decreased cell viability during the normal observation time was found to come from the decrease of cell number instead of the decrease of average viability per cell. The PDA coating led to less cell global migration yet more local motility of cells. Based on the concept of ‘background adhesion’ of cells on a surface without significant motifs of specific cell adhesion, we supposed that cells adhered to the PDA coating better, which influenced mobility and eventually proliferation. Hence, the cell behaviors on the PDA coating are reasonable, albeit a bit complicated.

## Graphical Abstract

**Figure:**
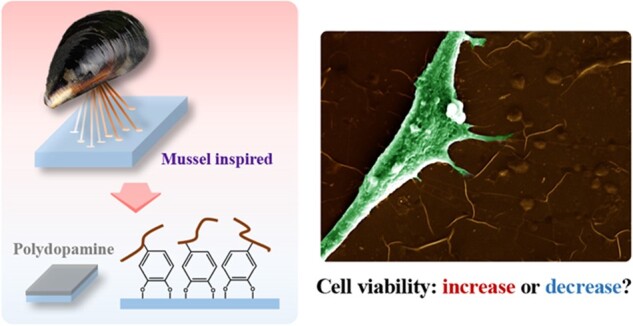

## Introduction

For medical devices and drug delivery vehicles, surface modification is an efficient strategy to adjust cell–material interactions [1–3]. A material surface can be modified via physical and chemical ways [4]. As the chemical way is concerned, the key technique is how to tightly bind or strongly adsorb a functional moiety on a matrix, which is dependent upon the chemical properties of both the substrate and the functional chemicals [5]. A breakthrough occurred in 2007 when Messersmith’s group reported a universal approach to enable binding between various substrates and various functional moieties mediated by polydopamine (PDA), a mussel-inspired polymer in the journal *Science* [6]. Since the publication of the *Science* 2007 article, PDA has been a superstar molecule to mediate biofunctionalization of many substrates [7–9], and is still very hot according to our literature retrieval. Using ‘polydopamine’ as the keyword in the Web of Science, we find only one paper before 2007 but about 12 000 publications after 2007, in which about two-thirds are related to biomedicine and surface modification; and the latest 2.5 years have witnessed about 3500 publications, as seen in Supplementary Fig. S1. The chemical structure of PDA is presented in Supplementary Fig. S2. PDA coatings have been applied to facilitate highly efficient bindings of growth factors, bioactive proteins or functional peptides on bioinert substrates for realizing the bio-related functions of materials or regulating cell behaviors [10–13]. Among these strategies, bioactive modifications by anchoring bioactive substances assisted by PDA has been quite popular to enhance or regulate proliferation, migration and differentiation of many types of cells and eventually promote the performance of biomaterials and medical devices [14–17].

**Figure 1.**
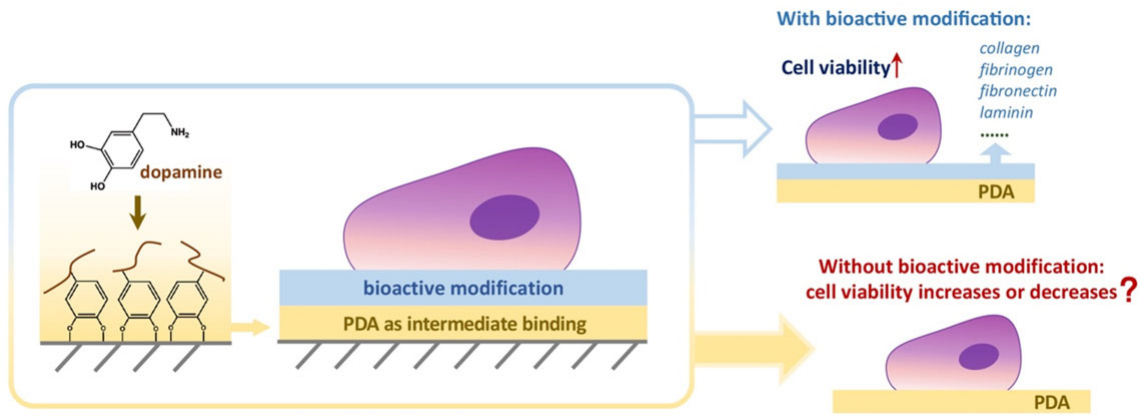
Schematic illustration of surface modification by PDA and its potential effects on cells. A question is addressed by us—is PDA itself beneficial for cells?

According to the standard of biological evaluation of medical devices offered by the International Organization for Standardization (ISO 10993-5: 2018), acceptable cytotoxicity and adequate cell viability of any biomedical material should be accessed following the ISO protocol and promised as a precondition [18]. When PDA worked for mediating further bioactive modification, cell viability was undoubtedly improved [19–21]. However, a few years ago, our group observed the decrease of cell viability on titanium with a PDA coating (data not shown). We then tried to compare our results with those published data by others. To our surprise, it is unbelievable that the cell viability of PDA itself has rarely been reported. Only a few data concern the viability of cells on a PDA layer [22–24], and there is no unified description. We have spent quite a few years confirming the repeatability of this puzzling phenomenon for a superstar molecule and giving a preliminary interpretation. After the efforts of successively three graduates and other group members, we are now sure that our description of the ‘negative’ phenomenon is true. In order to make the conclusion more universal, the data reported in the present first publication focused on the effects of the PDA itself on cell viability were collected using conventional cell culture plates (TCPs) composed of medical-grade polystyrene as the substrate. The problem attacked in this article is schematically presented in Fig. 1.

Herein, PDA was deposited by oxidative polymerization of dopamine. Concentrations of dopamine for the PDA modification were set as 1 and 2 mg ml^−1^ on normal TCPs. The corresponding experimental groups are denoted as PDA-1 and PDA-2, respectively. Cell viability was detected with thiazolyl blue tetrazolium bromide (MTT) or its improved way called cell counting kit 8 (CCK-8) following ISO 10993-5: 2018. These two methods share similar detection principle. Owing to being more convenient albeit more expensive, CCK-8 has been the predominant approach, and its principle is schematically presented in Supplementary Fig. S3. Enzyme reactions were used to reflect the viability of cells on a surface or the toxicity of a substance to cells. The CCK-8 reagent contains WST-8, which is reduced to a highly water-soluble yellow formazan dye by dehydrogenase in cell mitochondria mediated by an electron carrier 1-methoxy-5-methylphenazinium methyl sulfate. The optical density (OD) of the formazan dye quantifies the concentration of the ‘product’ of the enzyme reaction and thus the total enzyme activities under a given and sufficient concentration of ‘substrate’. So, the total viability of the detected cells was characterized indirectly via CCK-8.

Human bone marrow mesenchymal stem cells (hMSCs), a valuable cell source for tissue engineering and regenerative medicine, were employed as the default model cell type. Most of data of cell viabilities resulted in hMSCs on TCPs with and without the PDA coating. Some data came from other cell types including human umbilical vein endothelial cells, human foreskin fibroblast (HFF), a typical human cervical cancer cell line (HeLa), human lung cancer alveolar basal epithelial cells (A549) and other substrates including Ti6Al4V representative of metals, glass representative of nonmetallic inorganics and plasma-treated polydimethylsiloxane representative of polymers. Besides cell adhesion and proliferation, cell motility on a PDA coating was examined. The results indicate that the so-called abnormal phenomenon of cell viability on a PDA coating is conditional, reasonable and not harmful for the potential application of this superstar molecule.

## Materials and methods

### PDA modification on TCPs

We used polystyrene tissue culture plate (Corning Inc., Model: Corning Costar 3506/3513/3524/3548/3896) as substrates. No extra pre-treatment was given to the substrates except for PDA modification. According to the instructions offered by Corning, the purchased TCPs (the so-called normal or virgin TCPs in this article) have all been subject to tissue culture treatment (usually via plasma surface modification) for suitable cell adhesion.

For PDA modification, dopamine hydrochloride (Sigma) was dissolved in Tris-HCl (10 mM, pH 8.5). Dopamine solutions with concentrations of 1 and 2 mg ml^−1^ were added on TCPs at room temperature. After keeping reaction overnight, the dishes were thoroughly rinsed with deionized water to remove the unattached dopamine and then blown dried. The modified substrates denoted as PDA-1 and PDA-2 resulted from with 1 and 2 mg ml^−1^ of dopamine solutions, respectively. The untreated TCP was set as the blank control.

### Cell culture

hMSCs were purchased from Chinese Academy of Science, and cultured in a humidified incubator (Binder, Germany) containing 5% CO_2_ at 37°C. Complete cell culture medium was composed of alpha minimum essential medium (Invitrogen) supplemented with 10% fetal bovine serum (Sciencell), 1% penicillin–streptomycin solution and 1% glucose (Gibco). The culture medium was regularly replaced with fresh complete cell culture medium every 48 h during the culture period.

### Cell seeding and CCK-8 assay

TCPs were sterilized with 75% ethanol and then washed with sufficient Phosphate buffered saline (PBS) solution. After seeding hMSCs for a given time, cell viability was tested by CCK-8 kit (Dojindo Laboratories, Japan). The seeding density should be appropriate. For instance, the cell density was usually no higher than 4000 cells per well for 96 well plates, and the initial density should be further reduced for tests permitting a long-term cell proliferation. The amount of CCK-8 reagent accounted for 10% of the total volume of the medium; after incubating for 2 h, the absorbance at 450 nm was determined, following the Dojindo protocol.

### Cell adhesion and immunocytochemistry assay

After cells were seeded to PDA-1, PDA-2 and the untreated TCP for 24 h or 72 h, the plates were rinsed with PBS for three times to remove dead and non-adherent cells. The adherent cells were fixed with 4% paraformaldehyde. Afterwards, the F-actins of cells were stained in red with phalloidin-Rhodamine (Abcam, USA), and nuclei were stained in blue with 4′,6-diamidino-2-phenylindole (DAPI, Beyotime, China). Fluorescence images were taken with an inverted fluorescence imaging system (Carl Zeiss, Germany).

### Cell proliferation characterization

First, we used total DNA content of cells to reflect the total number of cells via the approach of fluorometric analysis in QM40 fluorescence lifetime spectrometer (PTI, USA). As such, we prepared calf thymus DNA (1 mg/ml) and Hoechst 33258 (200 ng/ml) in sodium phosphate buffer (2 mM Na_2_EDTA, 0.05 M sodium phosphate, pH 7.0), then prepared DNA standard solutions (200–2000 ng/ml) with this buffer. Samples and the DNA standard solution were kept in a 37°C water bath for 20 min and quickly cooled down to –80°C for another 30 min. This step was repeated for at least three times for complete DNA release out of cells, and eventually a transparent solution was obtained. Hoechst 33258 (Sigma) was added to mark the DNA for 30 min. The fluorescence emission at 455 nm was measured with an excitation wavelength of 350 nm.

To further confirm the proliferation ability of cells on each substrate, we carried out 5-ethynyl-2′-deoxyuridine (EdU) incorporation assay. EdU worked as an analogue of thymidine and was incorporated in DNA strands to sign new-proliferating cells. Cell-light EdU cell proliferation kit (RiboBio Inc, China) was applied in this work for identifying cells that were undergoing DNA synthesis during exposure to EdU. We added 50 μM of the EdU working solution into culture medium after stable adhesion of cells. After 2 h, cells were fixed with 4% paraformaldehyde and incubated with 2 mg/ml amino acetic for 5 min. Cells were permeabilized with Triton X-100 (0.1% v/v) for 10 min, and then strained by 100 μl of Apollo 488 solution for 30 min. DAPI was used to stain cell nuclei.

We also performed Western blot of Ki67, a proliferation-related nuclear protein. Cell lysates were harvested using RIPA lysis buffer (Service bio), and then denaturized at high temperature to perform sodium dodecyl sulfate-polyacrylamide gel electrophoresis (SDS-PAGE). SDS-PAGE gels (8 wt%) were used to load the samples to separate the proteins, then transferred onto poly(vinylidene fluoride) membranes. The membranes were incubated with mouse monoclonal Ki67 primary antibody (Abcam) at 4°C overnight, then incubated with IRDye 800CW-conjugated goat anti-mouse second antibody (Abcam) at room temperature for 2 h. We used LI-COR imaging system (LI-COR Biosciences) to visualize the bands and then analyzed the bands via the free software ImageJ.

### Cell cycle analysis

We recorded the growth curve to reflect quantitatively the process of cell proliferation, according to which cell doubling time could be further calculated. As such, we cultured hMSCs in logarithmic growth phase in the complete medium on TCP, PDA-1 and PDA-2. We used CCK-8 assay to obtain OD values during the 12 days. Cell average doubling time of cells was calculated from:

$$\mathrm{Average}\;\mathrm{doubling}\;\mathrm{time}=\left(t-t_{0}\right)\;\mathrm{lg}2/\left(\mathrm{lg}N_{t}-\mathrm{lg}N_{0}\right)$$

Here, *t* and *t*_0_ represent the end time and start time of cell culture, and *N*_0_ and *N_t_* are the numbers of cells or the corresponding ODs at the beginning and the end, respectively.

The mitotic cycle of cells was also analyzed with flow cytometry. The hMSCs pre-cultured on PDA-1, PDA-2 and TCP were harvested by trypsinization, washed twice with ice-cold PBS and fixed in 75 wt% ethanol at 4°C for 2 h. After rinsed with PBS for three times, cells were resuspended in PBS (0.5 ml) containing also 100 μg/ml propidium iodide, 0.1 wt% Triton X-100, 0.0037% EDTA and 0.2 mg/ml DNase-free RNase. About 10 000 events were measured with an FACS SCAN Flow Cytometer (Becton-Dickinson, USA) for each sample to analyze the percentages of cells in G0/G1, S, G2/M using Moffit LT 3.0 analytical software.

### Random migration of single cells

We added an hMSC suspension on culture wells with the density of 2 × 10^4^ cells per milliliter. After cell seeding for 8 h, cells were rinsed with PBS, and nuclei were stained with Hoechst 33342 (Invitrogen) in blue. The dye solution was then replaced by the fresh complete culture medium. Continuous shooting of cell migration was achieved with a microscopic observation system (Observer Z1, Zeiss) equipped with a living cell culture device. ZEN software (Zeiss) was used to capture images with 5× objective every 30 min, lasting for 24 h.

To quantify cell migration, we input cell image sequences into ImageJ software to obtain the coordinates of each cell nucleus, which represented the position of real-time location of a cell. We defined ***r_i_*** (*i *=* *0, 2, …, *n*) as the position vector of a cell at time *t_i_*, in which *t*_0_ represents for the initial record time of each cell. In our study, the time interval to record two consecutive positions of each cell Δt was 0.5 h.

For a random walk, the contour length of the cell trajectory is dependent upon Δ*t* and thus not a constant. Stimulated by the classical treatment of Brownian motion by Einstein in 1905 [25], diffusivity *D* was introduced to quantify the ability of cell migration based on mean square displacement (MSD). The two-dimensional form is written as:

$$\mathrm{MSD}=\;{<{\left[r\left(t\right)-r\left(t_{0}\right)\right]}^{2}>}_{\mathrm{cell}}\;=4D\left(t-t_{0}\right)$$

### Cell local motility analysis

We suggested to employ temporal fluctuation of cell perimeter to quantify the local motility of a cell on a surface. For the measurement of cell perimeter of each time point, hMSCs were seeded to the substrate with the cell density of 2$\times 10^{4}$/ml, and nuclei of live cells were stained with Hoechst 33342 in blue. For cell motility analysis, the real-time observation of living cells lasted for 20 h with images captured every 10 min and imaged with 20× objective.

To measure cell perimeters, we input cell image sequences into ImageJ software and employed the built-in graphic plug-in component to calculate the time-dependent perimeters of the cells. We defined the ratio of the standard deviation of cell perimeter to the average perimeter as a fluctuation index to quantify the extent of local motility of cells on different substrates.

### Cell centrifugation assays

We cultured hMSCs on 35-mm glass bottom plates (ibidi, Germany) with the cell density of 25 000 cells in each plate for 8 h. Nuclei of live cells were stained with Hoechst 33342 in blue. Then the plates were inverted to replace the cap of centrifugal tube (Corning) and centrifugated at 2350 rpm (900 g) for 10 min. The number of cells before (*N*_0_) and after centrifugation (*N*_t_) were counted. The residual fraction *N*_t_/*N*_0_ reflected the capacity of cell adhesion on a surface to some extents.

### Statistical analysis

For cell adhesion and single cell migration, 10 independent experiments participated in statistical analysis for each group, and at least 30 cells were counted in each independent experiment. For CCK-8 assay, Western blot, flow cytometry and cell centrifugation assay, five independent experiments were settled for each group. For local cell motility analysis, three independent experiments participated in statistical analysis for each group, and in each independent experiment, at least 20 cells were counted. Statistically significant differences between any two of the PDA-1, PDA-2 and TCP groups were determined using one-way analysis of variance (ANOVA). A significant difference was acceptable when *P *<* *0.05. We denoted ‘*’ as *P *<* *0.05 and ‘**’ as *P *<* *0.01.

## Results

### Verification of viability decrease of cells on PDA-coated surfaces

**Figure 2.**
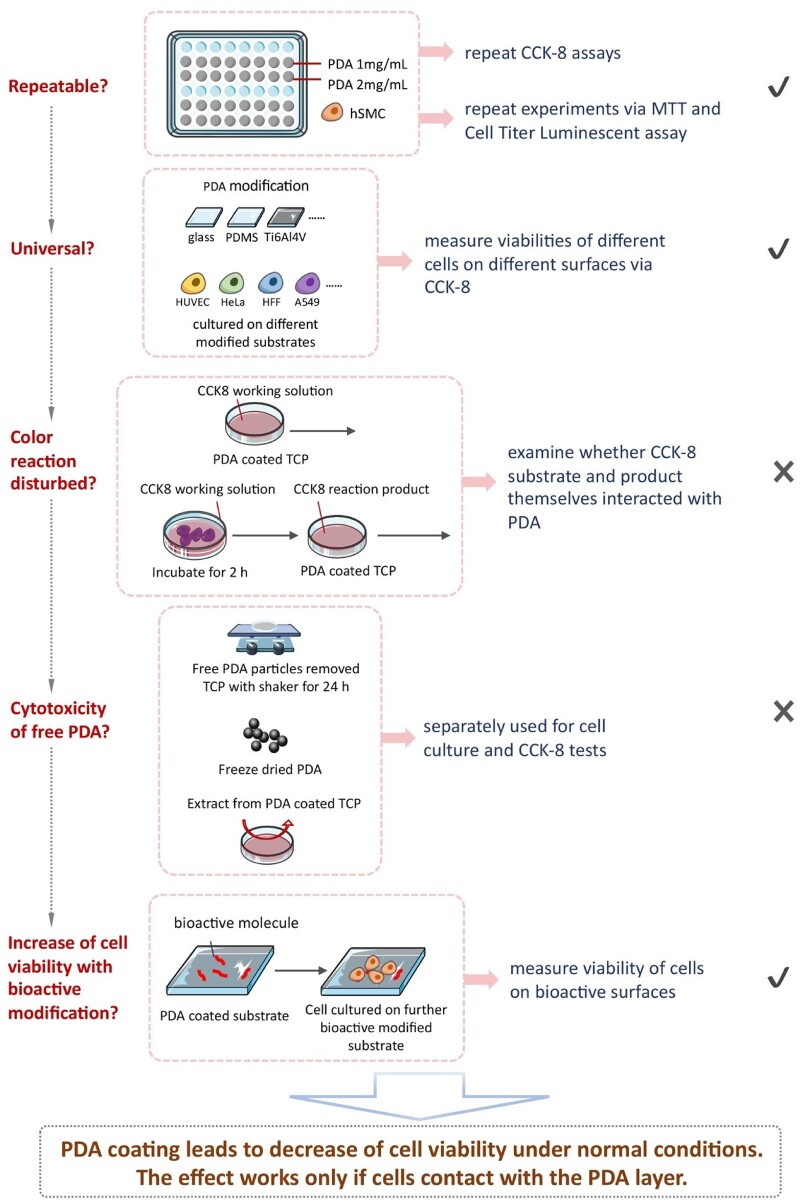
Schematic illustration of experimental strategies and processes to verify the decrease of cell viability on the PDA-modified surfaces in respect of virgin substrate. We elucidated that it was a universal and contact-dependent phenomenon.

Our group observed the decrease of viability of cells on titanium with PDA coating (data not shown and not published). Although about 12 000 papers about PDA have been published since the pioneering work by Messersmith’s group in *Science* 2007, this ‘negative’ viewpoint has not yet been set. So, we seriously treated this ‘abnormal’ phenomenon of a superstar molecule and spent several years to confirm or deny this conclusion. The group leader arranged group members from classes of different years to repeat the experiments and examined this phenomenon from different aspects. Some of the representative efforts are summarized in Fig. 2.

The experimental details are listed in Supplementary Table S1. The corresponding results are briefly summarized as follows:

First, we confirmed that the decrease of cell viability was repeatable. Then, we asked whether or not this was caused specifically by the CCK-8 characterization approach. So, we carried out a more classic way MTT and a more advanced way Cell Titer Luminescent assays. Both of them outputted the same trend as CCK-8. The results are shown in Supplementary Fig. S4.

We asked whether or not this was caused specifically by cell type or substrate. So, we examined different cell types on different substrates with and without PDA modifications. The results in Supplementary Figs S5 and S6 indicate that the decrease trend of cell viability on PDA-modified surfaces with respect to the virgin substrate kept well on different cell types and substrates. So, the conclusion is universal.

But the cell viability of PDA itself has rarely been reported according to our hard literature retrieval. In order to avoid any reckless disclosure of a bad news for a superstar molecule, we further asked whether or not there was any interference of the PDA to the substrate or product of the enzyme reaction underlying the CCK-8 test. We designed experiments as indicated in Supplementary Table S1. The results in Supplementary Fig. S7 rule out this possibility.

Then we asked whether or not some PDA nanoparticles joined in the cytotoxicity and thus led to the decrease of the cell viability on PDA coating. Supplementary Fig. S8 rules out this possibility. The viability was decreased after the cells were in contact with the PDA layer.

In spite of the decreased cell viability on PDA-modified surfaces, the further bioactive modification can well increase the total cell viability, as is well known. For instance, PDA-mediated covalent functionalization of collagen on a titanium alloy Ti–6Al–4V was confirmed to enhance the viabilities of HFFs and human immortal keratinocytes (HaCaTs) and promote biocompatibility with soft tissues [26]. In this article, this is demonstrated by a collagen coating on TCP mediated with a PDA layer to increase the total viabilities of hMSCs, as shown in Supplementary Fig. S9. Additionally, we have found that a fibrinogen modification of Ti–6Al–4V mediated by a PDA pre-coating significantly increased cell adhesion *in vitro* and biological sealing *in vivo*, resulting in well integration of a surface-modified implant with both soft and hard tissues in a rat model [27].

After the article was reviewed, one of professional referees suggested ‘trying a lower dopamine concentration and less coating time to further verify the conclusion’. The authors designed new experiments to modify the TCPs with different concentrations of dopamine in Tris-HCl (0.1, 0.5, 1 and 2 mg/ml) for different coating times (0.5, 2 and 24 h). According to the CCK-8 data shown in Supplementary Fig. S10, neither low dopamine concentration nor short modification time led to significant change of cell viability. Meanwhile, the decrease of cell viability occurred on surfaces treated by high-concentrated PDA for a sufficiently long modification time.

Hence, the unexpected ‘abnormal’ phenomenon of cell viability decrease was repeatable and versatile for varied cell types and initial substrates. The PDA coating led to a decrease of cell viability under normal culture and observation conditions, and this effect worked only if cells contacted with the PDA layer.

### Total cell viability and average cell viability

**Figure 3.**
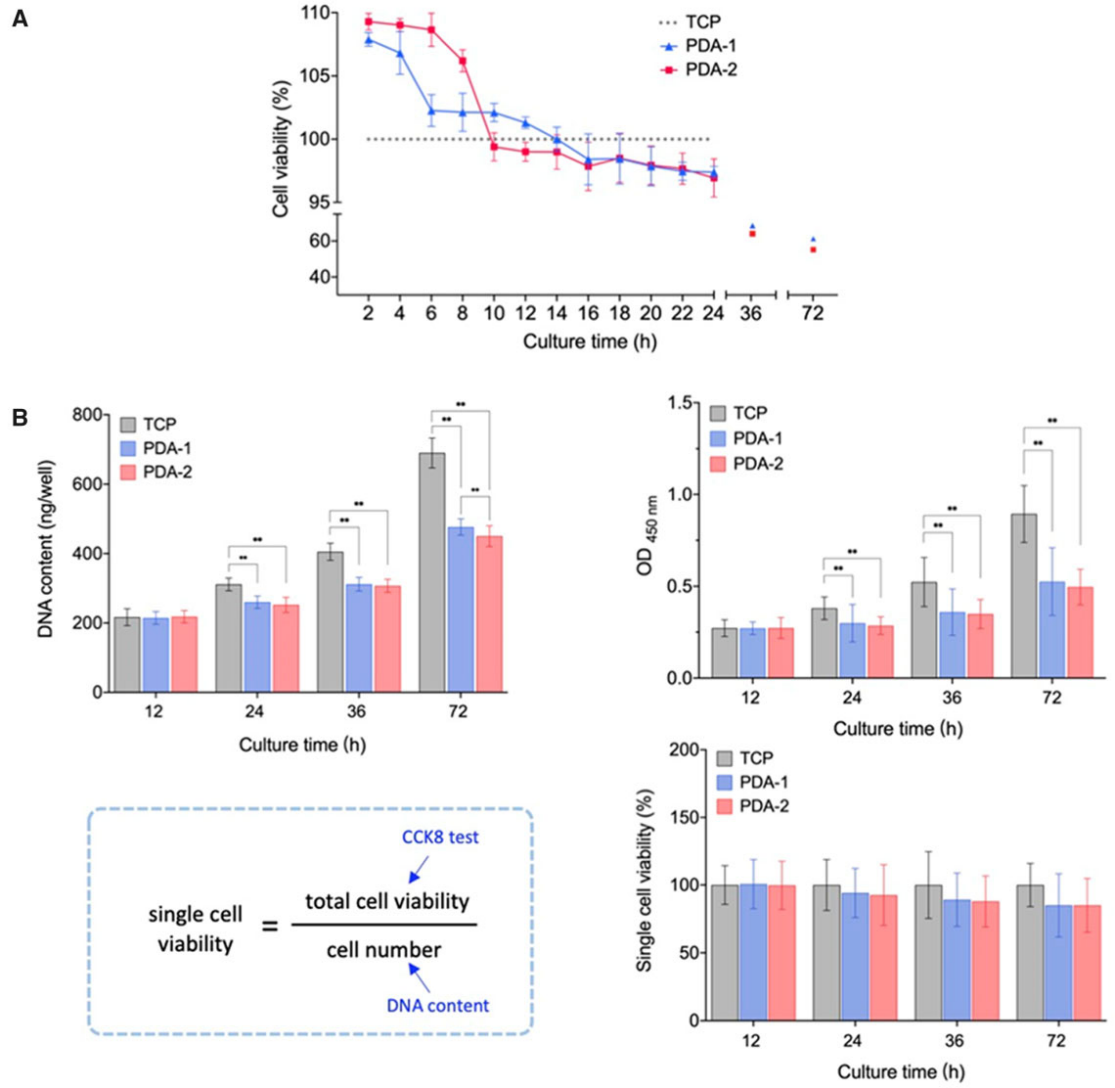
Finding of time dependence of relative viability of cells on PDA-modified surfaces with respect to the virgin substrate as demonstrated by hMSCs on TCP with and without PDA coating. (**A**) Total viability of hMSCs on modified surfaces during 72 h normalized by those on virgin TCPs at the corresponding time points. PDA-1 and PDA-2 mean the TCPs modified with 1 mg/ml and 2 mg/ml dopamine, respectively. For each group, *n *=* *7. (**B**) Examination of PDA coating on single cell viability. We measured total DNA contents of hMSCs per well with fluorometric quantification of the DNA-Hoechst 33258 complex reflecting the cell number and the OD values with CCK-8 reflecting the total cell viability. Their combination led to single cell viabilities at each indicated time point, which exhibited no significant difference among time points. Data are presented as mean ± standard deviation and analyzed by one-way ANOVA. For each group, *n *=* *7. Significant differences are marked with one asterisk (*P *<* *0.05) and double asterisks (*P *<* *0.01).

CCK-8 reaction depends on the indirect reduction of WST-8 by intracellular dehydrogenase. So, the result given by CCK-8 was the total cell viability in each assay unit. The data pertinent to Fig. 3 were obtained following ISO 10993-5: 2018, and thus the cell culture periods were among 24–72 h. Here, we tried to examine cell viabilities for a shorter time. Figure 3A shows the normalized total cell viabilities of hMSCs on TCP, PDA-1 and PDA-2. Different from the lower ODs at the standard detection time, we found that a PDA coating led to higher total cell viability than the virgin TCP in the early stage, particularly within the first half of the day.

We further examined viability per cell. As such, DNA content of each group during 72 h was determined by fluorometric quantification. According to the equation in Fig. 3B, single cell viability was calculated, which showed no significant difference among groups.

### Cell proliferation and cell cycle

**Figure 4.**
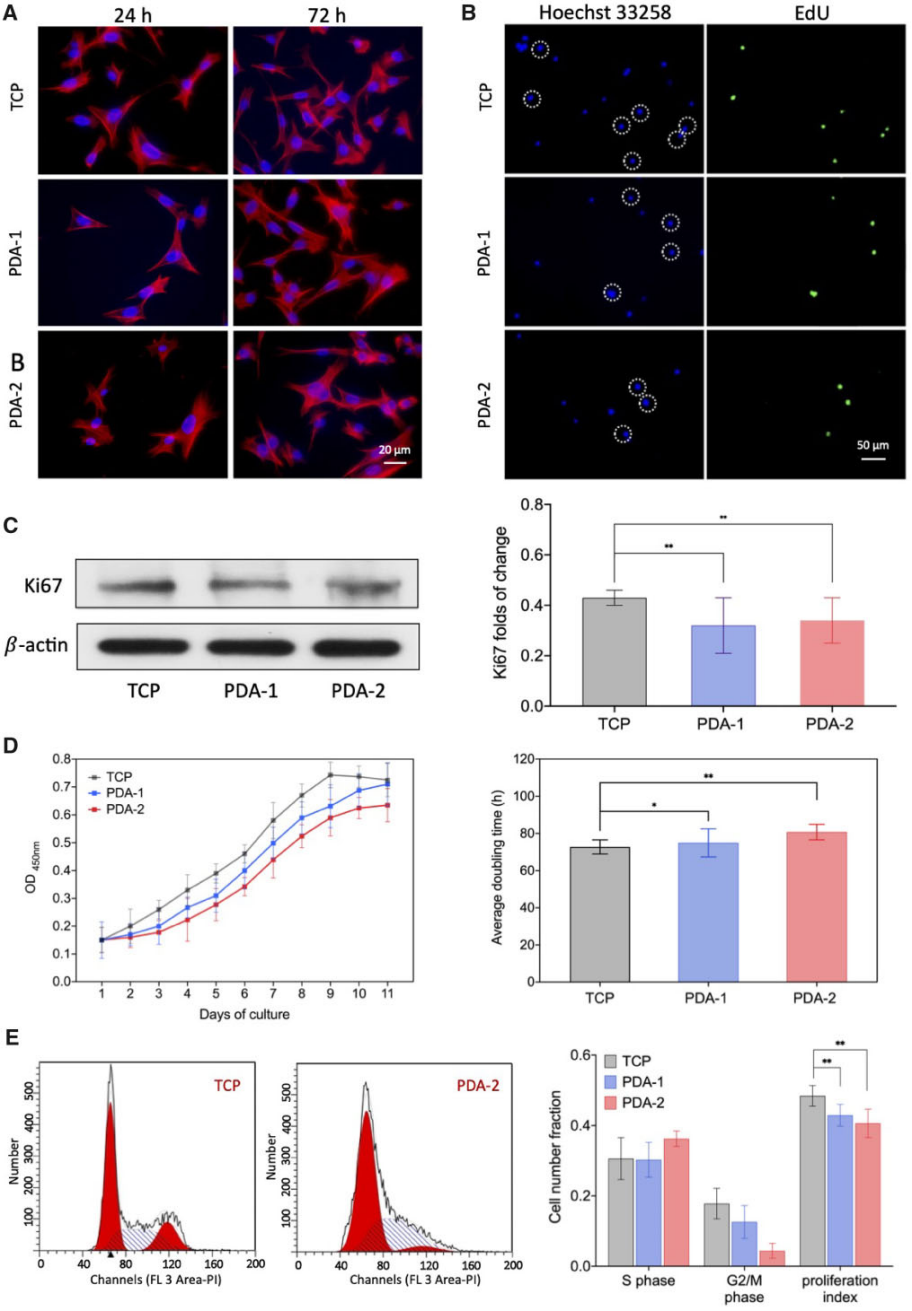
Examination of cell proliferation to reveal the slowing of proliferation of cells on PDA-modified surfaces. (**A**) Fluorescence micrographs of hMSCs on TCPs with or without PDA coating. After cultured for 24 and 72 h, the cells were stained with phalloidin to show F-actin (red) and with DAPI to show nuclei (blue). (**B**) Fluorescence micrographs of the nuclei of the cells on TCP and PDA-coated TCPs. Cells that were synthesizing DNA incorporated EdU into DNA strands (green). The cell nuclei were stained with Hoechst 33258 (blue). (**C**) Western blot of Ki67. For each group, *n *=* *5. (**D**) Growth curve of cells on TCPs with and without PDA coating, and the doubling times for each group calculated from total cell viabilities during the 9 days of culture. For each group, *n *=* *5. (**E**) Flow cytometry analysis and proliferous index statistics. The proliferation index is defined as the proportion of cells in S phase and G2/M phase to the total number of cells. For each group, *n *=* *5. Data are presented as mean ± standard deviation and analyzed by one-way ANOVA. Significant differences are marked with one asterisk (*P *<* *0.05) and double asterisks (*P *<* *0.01).

We stained hMSCs to visualize F-actin and nuclei. Typical cell images on TCP, PDA-1 and PDA-2 are shown in Fig. 4A. Micrographs, spreading areas and aspect ratios of the cells were analyzed, which exhibited no significant difference between different surfaces and the examined times (Supplementary Fig. S11). Nevertheless, the increase of cell density reflected cell proliferation from 24 to 72 h.

To investigate cell proliferation on TCPs with or without PDA coating, we employed EdU assay with its principle schematically presented in Supplementary Fig. S12. Newly synthesized DNA of the replicating cell was detected by the EdU incorporation assay to visualize newly proliferating cells nuclei in green. In Fig. 4B, fewer cell nuclei were labeled, indicative of inhibition of cell proliferation on PDA-modified surfaces.

We also carried out Western blot of Ki67. As illustrated in Fig. 4C, the protein as a marker of cell proliferation exhibited a significantly decrease after 24 h of culture of cells on substrates with PDA coating. Figure 4D shows the growth curve of cells, and doubling times were further calculated. Cell cycles were measured via flow cytometry with its principle schematically presented in Supplementary Fig. S13, and the proliferation index was given in Fig. 4E. All these results convinced that cell proliferation was alleviated on surfaces modified by PDA.

### Single cell migration on modified surfaces

**Figure 5.**
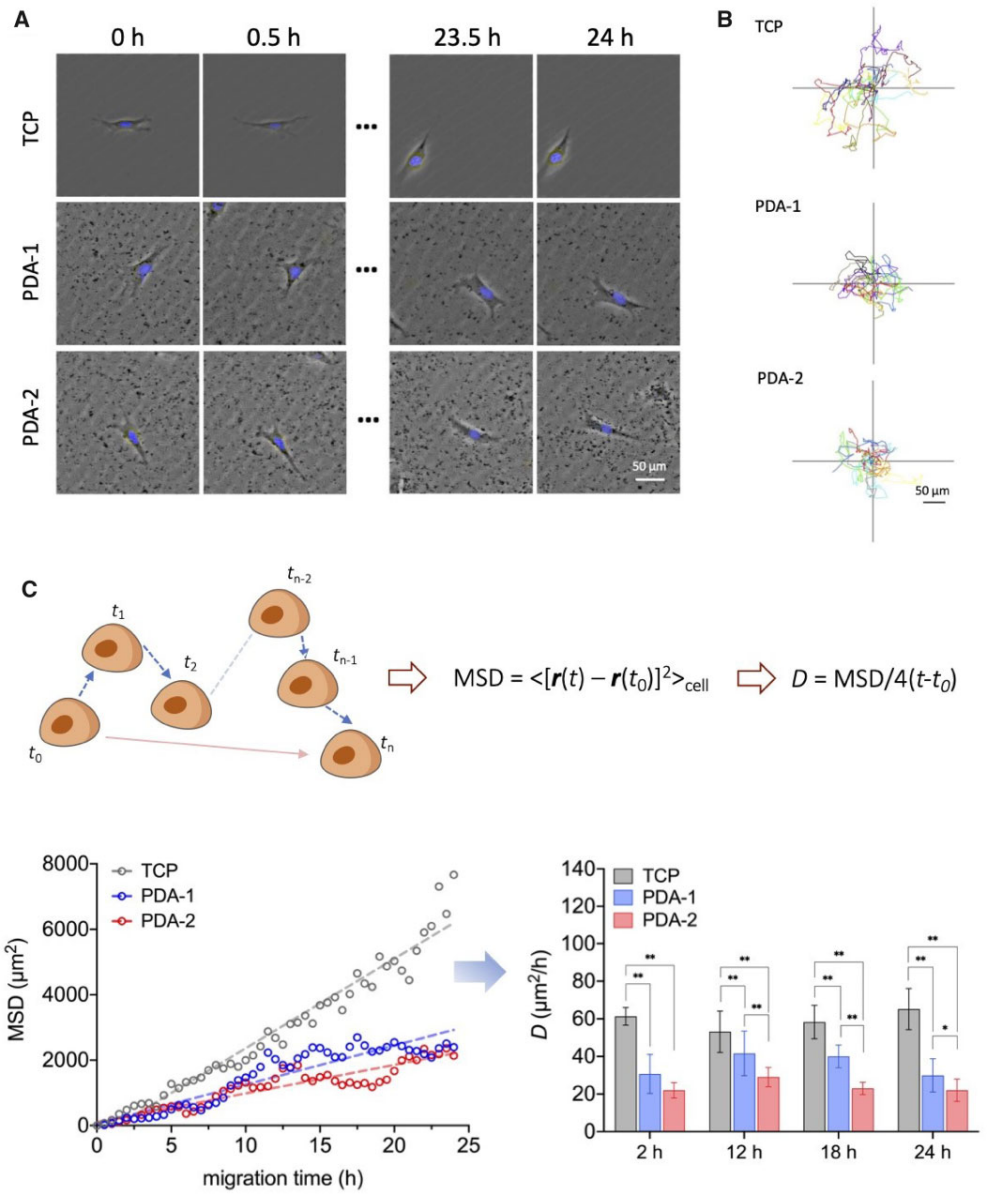
Cell migration of hMSCs on virgin TCPs and PDA-coated TCPs. (**A**) Live-cell time-lapse image of hMSCs at a low culturing density to demonstrate a single cell migration of hMSCs. Black spots came from deposition of PDA on the substrate. (**B**) Trajectories of random migration of single hMSCs. Each site represents the time-dependent position of the center of nucleus of a single cell. The position plots are labeled at the top left with numbers representing the average interparticle distances. Time interval of each two points was 30 min. (**C**) Statistics result of the tracking pathways of single cells with random-walk model. The mean square displacement MSD was calculated upon equal time interval. All the observation started at 12 h after cell culturing to ensure stable adhesion. The statistics were made based on the migration image series. For each group, *n *=* *40. Data are presented as mean ± standard deviation and analyzed by one-way ANOVA. Significant differences are marked with one asterisk (*P *<* *0.05) and double asterisks (*P *<* *0.01).

A living cell culture device was used for real-time observation of cells. Some typical micrographs and trajectories of single hMSCs during random migration are presented in Fig. 5A and B.

The cell migration looked like a random diffusion, and thus MSD might be linearly related to migration time, as shown in Fig. 5C. The calculated diffusivity *D* exhibited a significant difference between the virgin and modified surfaces.

### Local motility of cells on PDA-modified surfaces

**Figure 6.**
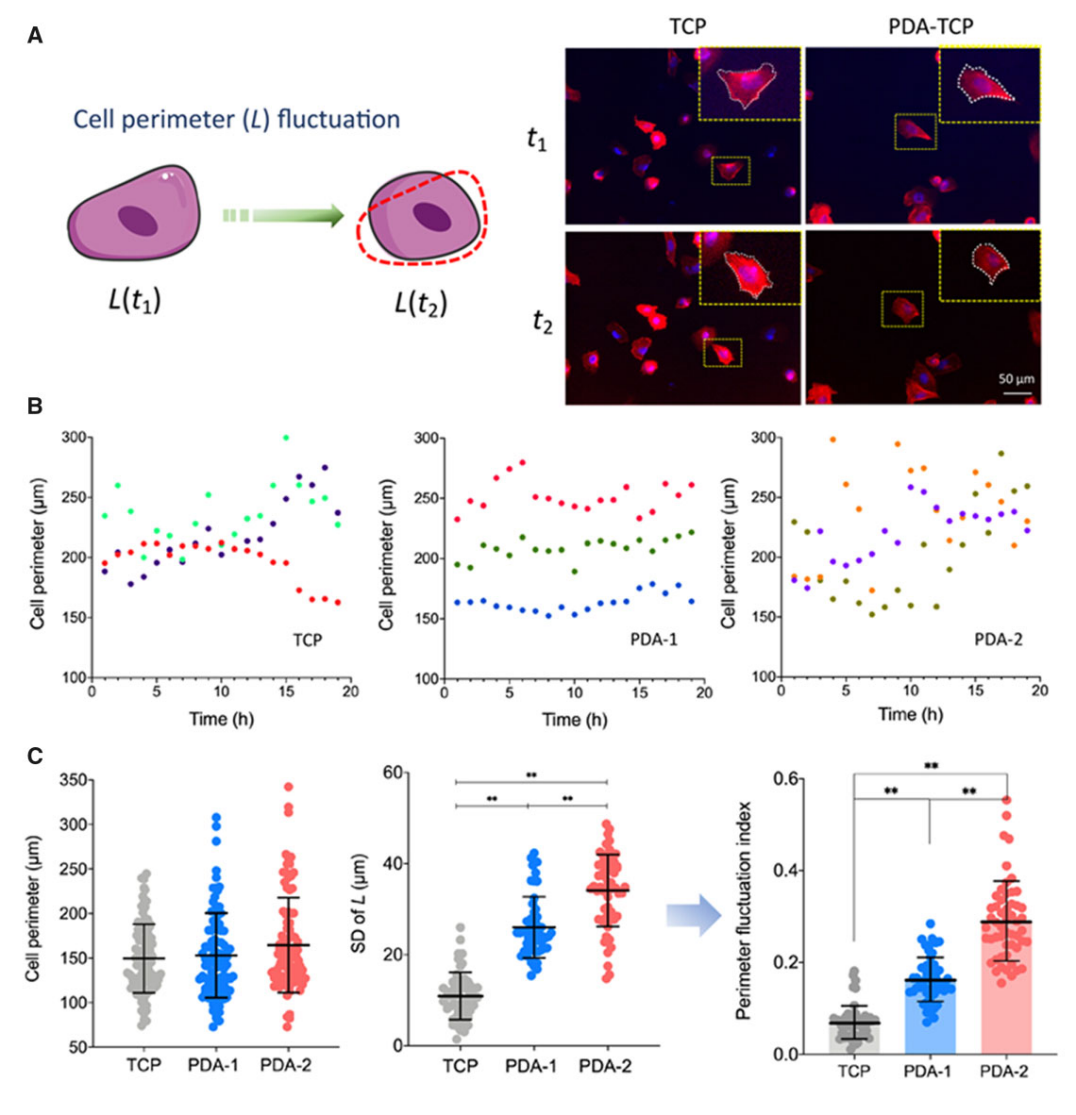
Local motility of hMSCs on virgin TCPs and PDA-coated TCPs. (**A**) Schematic graph of cell motility analysis via cell perimeter fluctuation and typical fluorescence micrographs from a live-cell time-lapse imaging system. (**B**) Demonstration of recorded fluctuation of cell perimeter with time (randomly selected three cells to show). (**C**) Distribution of cell perimeter, standard deviation (SD) and cell motility fluctuation index, which was defined as the ratio of the standard deviation of cell perimeter *L* to the corresponding average perimeter. For each group, *n *=* *95. Data are presented as mean ± standard deviation and analyzed by one-way ANOVA. Significant differences are marked with one asterisk (*P *<* *0.05) and double asterisks (*P *<* *0.01).

It is interesting that the PDA coating decreased the global cell migration but increased the local cell motility. We used the fluctuation of cell contour to reflect the local motility of cells. Cell perimeter was chosen to measure cell contour. We confirmed that this parameter reflected the differences among groups efficiently, as shown in Fig. 6A and B.

For a more quantitatively study on the cell perimeter fluctuation, we suggest a parameter termed ‘perimeter fluctuation index’, which is defined as the ratio of the standard deviation to the average perimeter of cells. According to Fig. 6C, PDA contributed to substantial fluctuation of cell perimeter along with time, despite no significant effect on the absolute perimeter of the cells.

### Adhesion capacity of cells on PDA-modified surfaces

**Figure 7.**
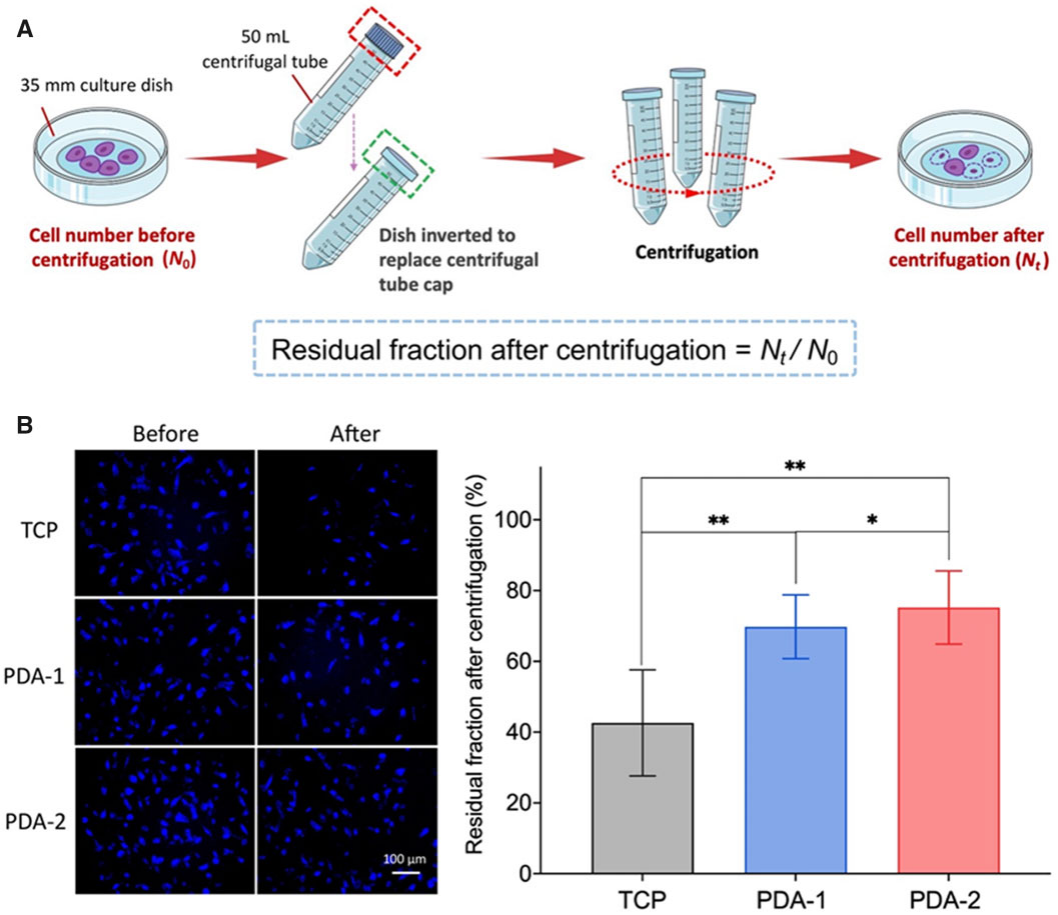
Semi-quantification of cell adhesion capacity measured by inverted centrifugation. (**A**) Schematic presentation of the principle. Cell centrifugation assays to exam adhesion extents of hMSCs on adhesive substrates coated by PDA. After culturing for 4 h, cells were inversely centrifugated for 15 min at 900 g. (**B**) Fluorescence micrographs and the calculated residual fraction of cells on surfaces before and after centrifugation. Cell nuclei were stained by Hoechst 33342 (blue) to enable counting of cell numbers and thus the fraction of residual cells after centrifugation. For each group, *n *=* *5.

We designed an experiment of inverted centrifugation to roughly estimate the adhesion capacities of cells on different surfaces. As shown in Fig. 7, residual fractions after centrifugation of cells on the PDA coatings were significantly larger than that on TCP. So, it is conclusive that the PDA-modified surfaces were more adhesive than the unmodified surface.

## Discussion

Understanding cell–material interactions constitutes a basis of biomaterial science and regenerative medicine [28–34]. It is the surface rather than the bulk that directly contacts with cells after implanting a biomaterial [35–37]. Surface modification is a cost-effective way to enhance the biofunction of the current biomaterials and a unique tool to develop next-generation bioactive materials [38–40]. Various surface modification approaches have been suggested [41–43], and the biomimetic PDA has been a superstar to mediate binding of a functional moiety to a substrate in a universal way [44–47]. While plenty of ‘good news’ have been reported about the effeteness of PDA to further bind biological active chemicals, the examination of the PDA itself on cells is relatively limited [48, 49].

Started from our unpublished finding of an ‘abnormal’ phenomenon of apparent cytotoxicity of the PDA via ISO 10993-5: 2018 to access cell viability of a biomaterial within usually 24–72 h, we have carried out a series of experiments to check the repeatability and understand the reason. As a result, we confirmed the decrease of cell viability when the cells directly contacted with the PDA coating; we also found the increase of cell viability at the early stage less than, for instance, 12 h. The time dependence and the strong ability of the PDA coating to bind biological substances might make the experiments complex and even lead to controversial descriptions of the PDA effects on cell viability. So, we have spent much time to well control the experiments, carefully examine the cell responses from different aspects and repeat our experiments many times. Although hMSCs and TCPs were employed as the default cell type and substrate, we also checked other cell types and substrates and have confirmed the universality of such an ‘abnormal’ phenomenon of a superstar molecule.

We further examined whether or not a PDA coating had any effect on cell cycle, cell migration and cell motility. It is interesting that while proliferation and global migration of cells were alleviated, the local motility of cells was promoted on the PDA. Now, we tried to explain the experimental results by proposing a unified rational to connect these experimental results.

### Cell proliferation relies on energy consumption under given energy supply

As was mentioned above, the OD value determined by CCK-8 reflects the total cell viability. According to Fig. 3B, single cell viability was not affected by the PDA coating. Thus, the change of total cell viability here was caused by that of the cell number or a relatively slower cell proliferation. The relatively slow proliferation of hMSCs on PDA-modified surfaces was related to cell cycle arrest.

As indicated in the initial paper of CCK-8 mechanism by Dojindo Laboratories, cell viability was proportional to the amount of formazan produced in the color reaction that was participated by dehydrogenase in live cells, mainly in the mitochondrion, the main organelle to supply energy to various cellular events [50]. As a participant in the tricarboxylic acid cycle, dehydrogenase plays a deterministic role for energy supply in cells. Cell proliferation is an energy-consuming process, and can be affected by viability of cells to some extent [51]. Here, we would like to emphasize that cell viability is conceptionally not identical to cell proliferation. While the dehydrogenase activity of a cell indicates the level of energy supply, the cell proliferation consumes only a part of generated energy. So, similar single cell viability in this study does not contradict with the different doubling times on virgin and PDA-modified surfaces. The fraction of energy consumption for cell proliferation relies also on the energy assignment, namely other cellular events which consume the supplied energy either.

### Cell motility must consume energy

Cell motility is a basic behavior of living cells and greatly associated with surfaces on which cells adhere to [52, 53]. Properties of surfaces such as chemical composition, mechanical properties or patterned cues influence cell responses [54–59]. According to our observations (Figs 5 and 6), PDA had opposite effects on migration and local motility of cells. It is interesting that the local contour fluctuation of cells on PDA was significantly larger than that on TCP.

Compared with cell migration which determined by the displacement of barycenter, local cell motility is a more primitive and direct way for the consumption of energy in cells [60, 61]. Thus, an enhanced cell motility on PDA is consistent with an alleviated cell proliferation.

### Background adhesion of cells on a surface

Cell adhesion is a key process among series of cellular events [62–64]. In general, a cell adheres to a surface through two basic ways: specific adhesion and nonspecific adhesion. Specific cell adhesion relies on bioconjugation between ligands and receptors [65]. By fabricating the adhesion ligand with a well-designed pattern, cell adhesion and its spreading area can be significantly affected [66]. Our group has ever adjusted the nanospacing of arginine–glycine–aspartate (RGD) peptides on pre-fabricated nanopatterns to regulate cell migration [67]. Besides specific adhesion, receptor-independent nonspecific adhesion is also important. As a generally adhesive substance like mussel protein, nonspecific adhesion on a PDA coating is worthy to reveal.

Herein, to better understand cells on PDA, we introduced a concept of ‘background adhesion’, which was considered to be independent of ligand–receptor binding, but associated with any nonspecific cell adhesion on a proper substrate. The enhanced background adhesion owing to the PDA coating led to a greater number of adherent cells and thus higher total cell viability in the early stage, as shown in Fig. 3A.

Cell adhesion is closely related to cell migration and motility. It has been known that cell migration constitutes two basic procedures, formation of leading edge and detachment of trailing edge [68–70]. The enhanced background adhesion by PDA coating is not beneficial for the detachment of trailing edge, and thus disfavors the change of center-of-mass of a cell, namely cell migration. Nevertheless, the less cell migration does not conflict with more frequent local trial and error and thus enhanced local motility. Previous publications confirmed this phenomenon by observation of live cells within a broad range of time scale, i.e. cell-shape fluctuations showed cooperative increasing associated with migration limitation [71, 72]. So, strong cell adhesion, reduced cell migration of center of mass and enhanced local cell motility are, although not necessarily co-occur, consistent with each other.

### Unified interpretation of cell behaviors on PDA-modified surfaces

**Figure 8.**
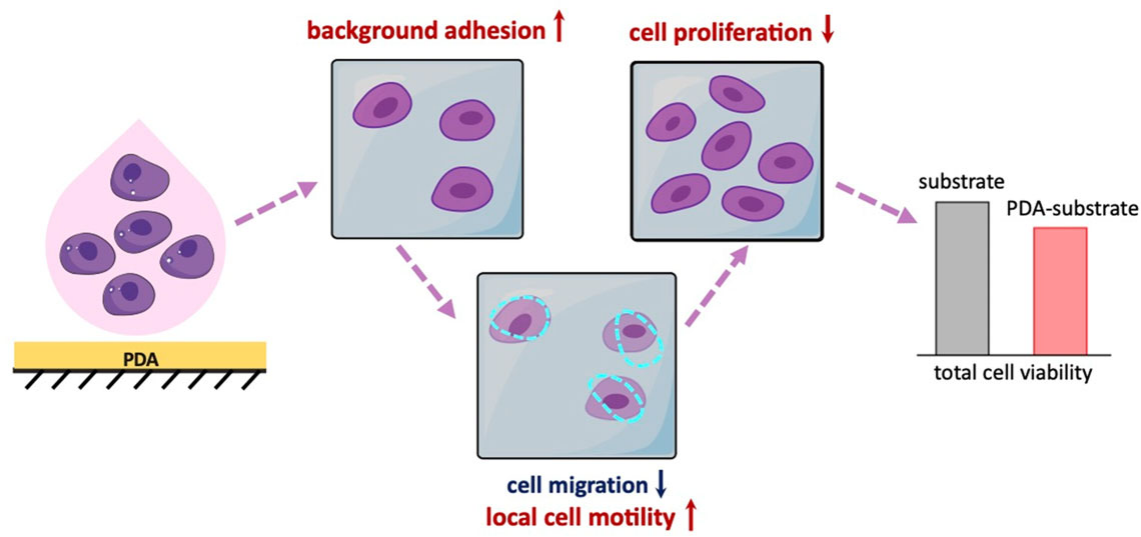
Schematic presentation of the reasons that the total cell viability decreases on a PDA-modified surface. PDA coating enhances ‘background adhesion’, which results in more cells on a modified surface in the early stage. However, local cell motility increases with stronger background adhesion examined in this study. The consumed energy leads to the inhibition of cell proliferation. Eventually, the experimental group with the PDA-modified surface exhibits lower total cell viability if examined at the normal culture time following ISO 10993-5: 2018 to access *in vitro* cytotoxicity of a biomaterial.

Many cell behaviors, including proliferation and motility, depend on energy supply and consumption. Once energy-dependent events intend to occur simultaneously, hierarchy of cellular energy consumption would be obeyed, for which some cell activities might be inhibited [73]. In this work, background adhesion of PDA resulted in the increase of local cell motility, which led to alleviation of another energy-consumed event, cell proliferation. The arrested cell cycle resulted in the less increase of cell number and relatively decrease of the total cell viability on PDA-modified surfaces, as schematically summarized in Fig. 8.

The present work has its limitations, for instance, lacking direct measurements of energy consumption. Despite of that, as the first paper focused on the PDA coating itself on cell behaviors, we examined cell adhesion, cell motility, cell proliferation and eventually unified these events in a rational way.

## Conclusions

This article reports a ‘negative’ result about a superstar molecular PDA, namely, the decrease of cell viability of a PDA coating upon ISO 10993-5: 2018, and elucidates a unified understanding. The decrease of the total cell viability arose from the alleviation of cell proliferation, which was reflected after 24–72 h incubation as suggested by the ISO. Actually, more cells adhered initially to the modified substance, which resulted in the increase of cell viability in the early stage. The PDA coating led to an enhanced ‘background adhesion’ of the modified surface to cells as well as many other biologically active substances. The background adhesion resulted in restricted cell migration, namely, the net movement of center of mass, but in enhanced local motility of cells. The cell motility might compete with cell proliferation as energy consumption is concerned, which has not yet been confirmed by any direct experimental evidence and thus calls for further investigation. Anyway, not only for PDA but also for any other surfaces with similar properties, the concept of background adhesion can establish a possible connection between adhesion, motility and proliferation of cells on a PDA-modified substrate. The decrease of cell viability following the ISO cannot deny the biocompatibility of PDA at all. Plenty of previous publications have illustrated that PDA is able to mediate binding of various biologically active substances to various matrixes in a biosafe way. In the present work, our objective and rational insight of the PDA itself on various cell events might stimulate further investigations and applications to take advantage of the complex and interesting cellular responses of this superstar molecule in the future.

## Supplementary data

Supplementary data are available at *REGBIO* online.

## Funding

This work was financially supported by the National Natural Science Foundation of China (grants No. 52130302 and 21961160721).

*Conflicts of interest statement*. None declared.

## Supplementary Material

rbac078_Supplementary_Data
