# Supplementary material for: Is polydopamine beneficial for cells on the modified surface?
Source: Regen Biomater. 2022 Oct 13;9:rbac078. doi: 10.1093/rb/rbac078 (PMC9610633; doi:10.1093/rb/rbac078)
Supplement: rbac078_Supplementary_Data [file rbac078_supplementary_data.pdf]

Supporting Information

Is polydopamine beneficial for cells on the modified surface?

*Yue Yu, Xiuli Wang, Yi Zhu, Yingning He, Hongrui Xue, Jiandong Ding**

State Key Laboratory of Molecular Engineering of Polymers, Department of Macromolecular Science, Fudan University, Shanghai 200438, China.

*Correspondence should be addressed to J.D. DING. E-mail: jdding1@fudan.edu.cn

This PDF file includes Supplementary Text, Figs. S1 to S13, Tables S1, which are shown in sequence mentioned in the main manuscript.

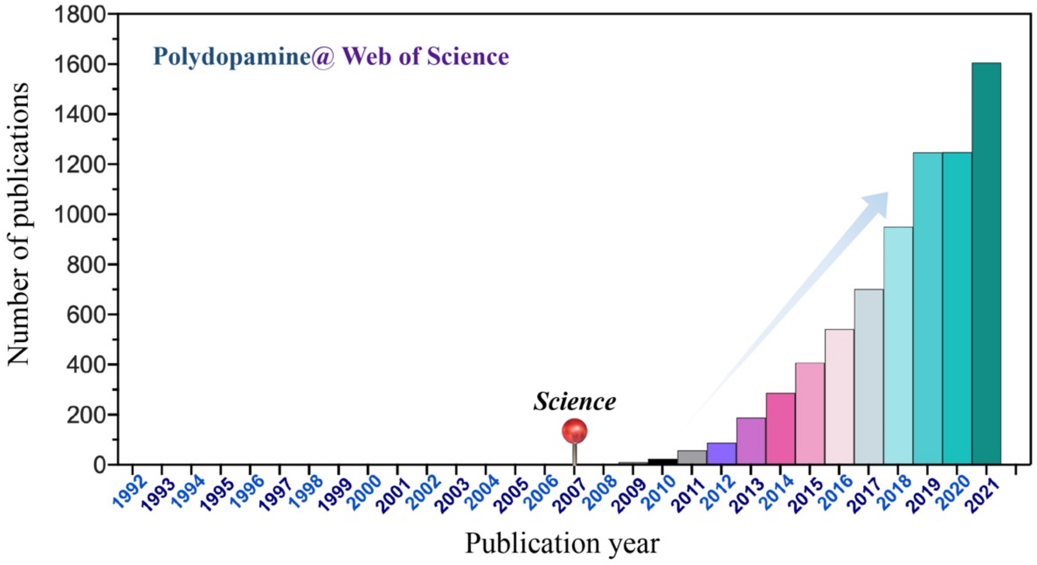

Fig. S1. Publication statistics about polydopamine for biomedical applications during the latest 30 years (15 years before 2007, and 15 years after 2007).

Source: Web of Science

Keywords: polydopamine. Output: 11977 publications. Only 1 publication appears during the 15 years (1992-2006) before 2007, one publication (Haeshin Lee, …, Phillip B. Messersmith*, *Science* **2007,** *318* (5849), 426-430) in 2007, but 11975 publications during the 15 years after 2007.

Keywords: polydopamine AND (cell OR biomaterial OR surface modification OR coating OR biomedical). Output: 8024 publications. No publication before 2007, one publication (Haeshin Lee, …, Phillip B. Messersmith*, *Science* **2007,** *318* (5849), 426-430) in 2007, but 8023 publications during the 15 years after 2007. Shown in the histogram are yearly publications till 2021, since the datum in 2022 is still incomplete and thus not shown. The summation of 2020, 2021, and the first half of 2022 reads 3518.

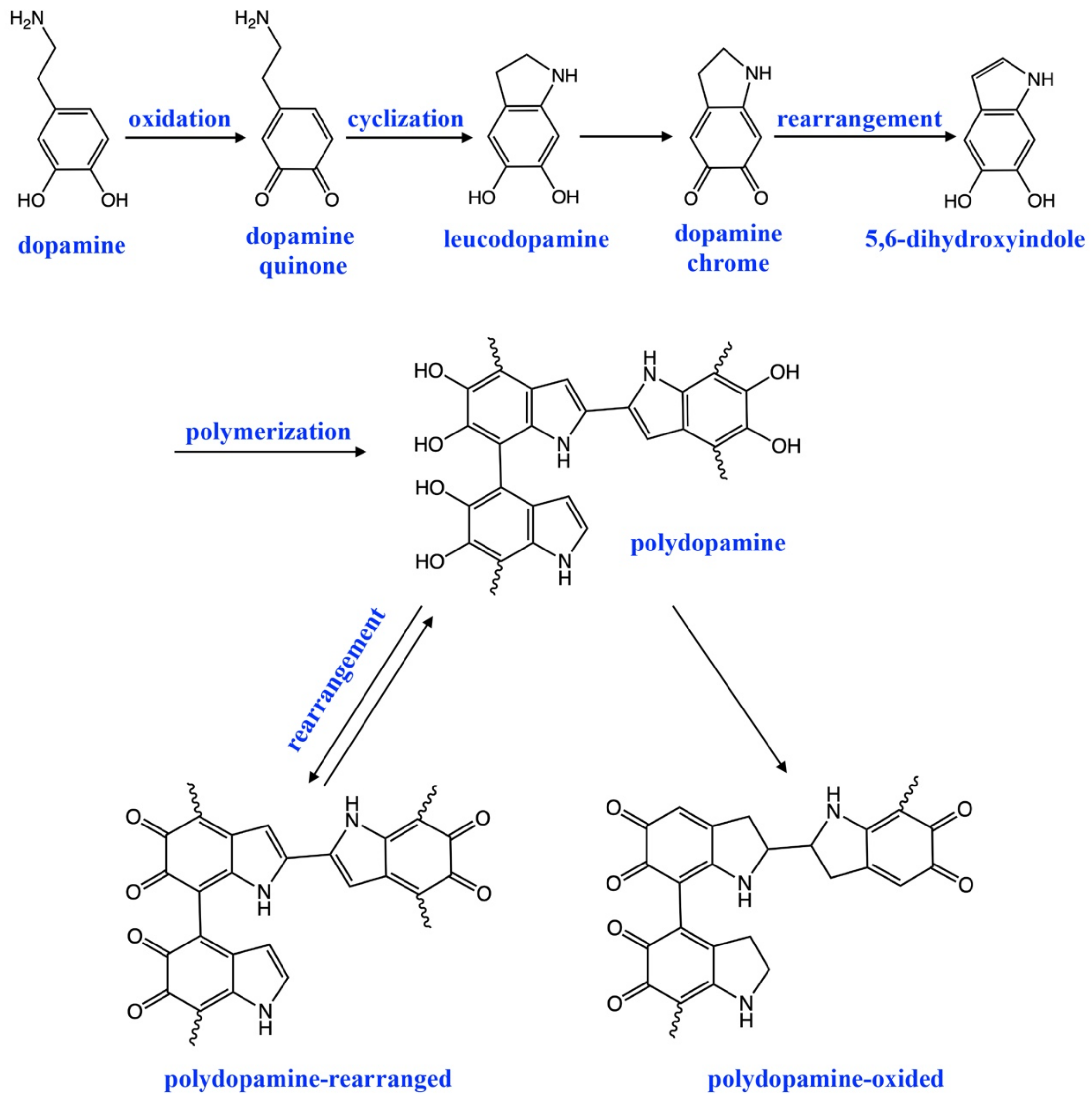

Fig. S2. The synthesis route of polydopamine (PDA) from dopamine, and the chemical structures of the oxidation or rearrangement products of PDA.

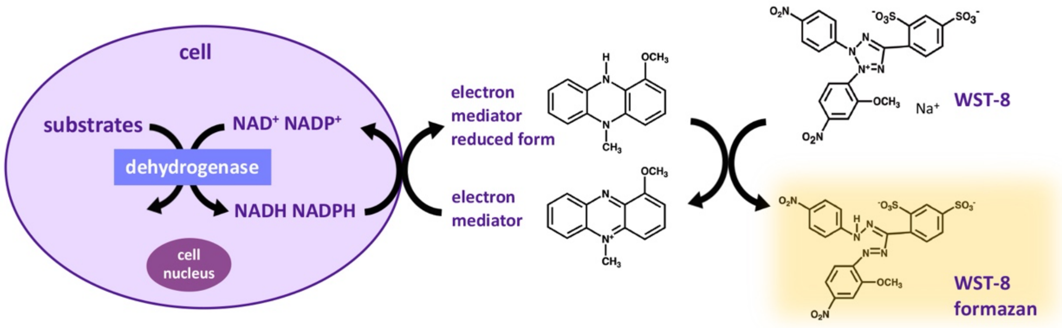

Fig. S3. Schematic diagram of the principle of CCK-8 test. The CCK-8 reagent contains WST-8, which is reduced to a highly water-soluble yellow formazan dye by dehydrogenase in cell mitochondria under the action of an electron carrier 1-methoxy PMS. The number of formazan dye produced is proportional to the number of living cells. This characteristic can be used to access cell proliferation and cytotoxicity of a biomaterial.

Table. S1. Verification strategies and results of possible factors that may be related to the decrease of total viability of hMSCs on PDA-coated TCPs compared with untreated TCP.

	**Possible factors to be confirmed or ruled out**	**Verification strategies**	**Results**	**Conclusions**
**Repeatability of the "abnormal" phenomenon of decreased cell viability on PDA-modified surface**	Abnormal results caused only by experimental errors	1. Repeat experiments (7 parallel samples for each group) for at least five times.<br>2. CCK-8 tests and statistics.	The same trend.	**Cell viability was surely decreased on PDA-coated surfaces at normal measurement time.**
	Abnormal results caused only by a specific experimental method	1. Use MTT test and CellTiter-Lumi™ Plus Luminescent Cell Viability Assay Kit instead of CCK-8 test.<br>2. Statistics and comparison.	The same trend as tested via CCK-8 test. **[Fig. S4.]**	
**Universality of the regulation**	Abnormal results caused only by a specific cell type	1. Culture different cells parallelly on TCP and PDA-coated surfaces.<br>2. CCK-8 test and statistics.	The same trend for different cell types. **[Fig. S5.]**	
	Abnormal results caused only by a specific substrate	1. Modify different materials including Ti6Al4V, glass, PDMS with PDA.<br>2. Culture cells on those materials.<br>3. CCK-8 tests and statistics.	The same trend for different substrates. **[Fig. S6.]**	
	Interaction between the *product* of the CCK-8 reaction and PDA on the color	1. Culture cells on TCP for 24 h.<br>2. Change medium into 10% CCK-8 (working solution) and incubate with cells for 2 h.	No significant difference between groups. **[Fig. S7.]**	

**Elimination of interference in test method**	reaction of CCK-8 test	3. Take the medium<br>4. Incubate the medium with TCP and PDA-coated TCPs (free of cells) for 2 h<br>5. Measure OD for each group.		
	Interaction between the *substrate* of the CCK-8 reaction and PDA on the color reaction of CCK-8 test	1. Incubate the medium containing 10% CCK-8 (working solution) with TCP and PDA-coated TCPs for 2 hours.<br>2. Measure OD for each group.	No significant difference between groups. **[Fig. S7.]**	
**Exclusion of non-contact factors**	Free PDA particles in culture medium	1. Prepare and freeze-dry PDA particles.<br>2. Culture cells in the presence of PDA particles.<br>3. CCK-8 test.	No significant difference between groups under normal concentrations, although excess particles causes cytotoxicity. **[Fig. S8.]**	**Decrease of cell viability was related to the direct contact between cells and PDA-coated surfaces.**
	Free PDA particles on PDA-coated surfaces	1. Prepare PDA-coated TCPs, wash off potential PDA particles in a decoloring shaker overnight.<br>2. Culture cells on surfaces surely without free PDA particles.<br>3. CCK-8 test.	Cell viability decreased similarly on PDA-modified surfaces without PDA particles. **[Fig. S8.]**	
	Extracts accumulated during culture	1. Prepare PDA coating, and prepare extracts using	No significant difference	

		complete culture medium.<br>2. Cell culture in the presence of the extracts.<br>3. CCK-8 test.	between groups.<br>**[Fig. S8.]**	

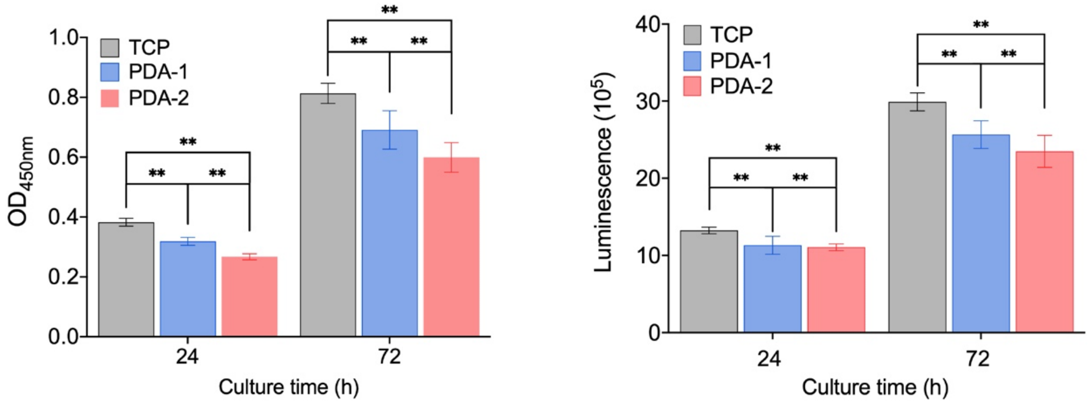

Fig. S4. The total cell viability of hMSCs on TCPs or PDA-coated TCPs via the other two test methods different from the default method CCK-8. The left was from the MTT assay, and the right was given by the CellTiter-Lumi™ Plus Luminescent Cell Viability Assay Kit. Data are presented as mean ± standard deviation and analyzed by one-way ANOVA. For each group, n = 5. Significant differences are marked with one asterisk ($p < 0.05$) and double asterisks ($p < 0.01$).

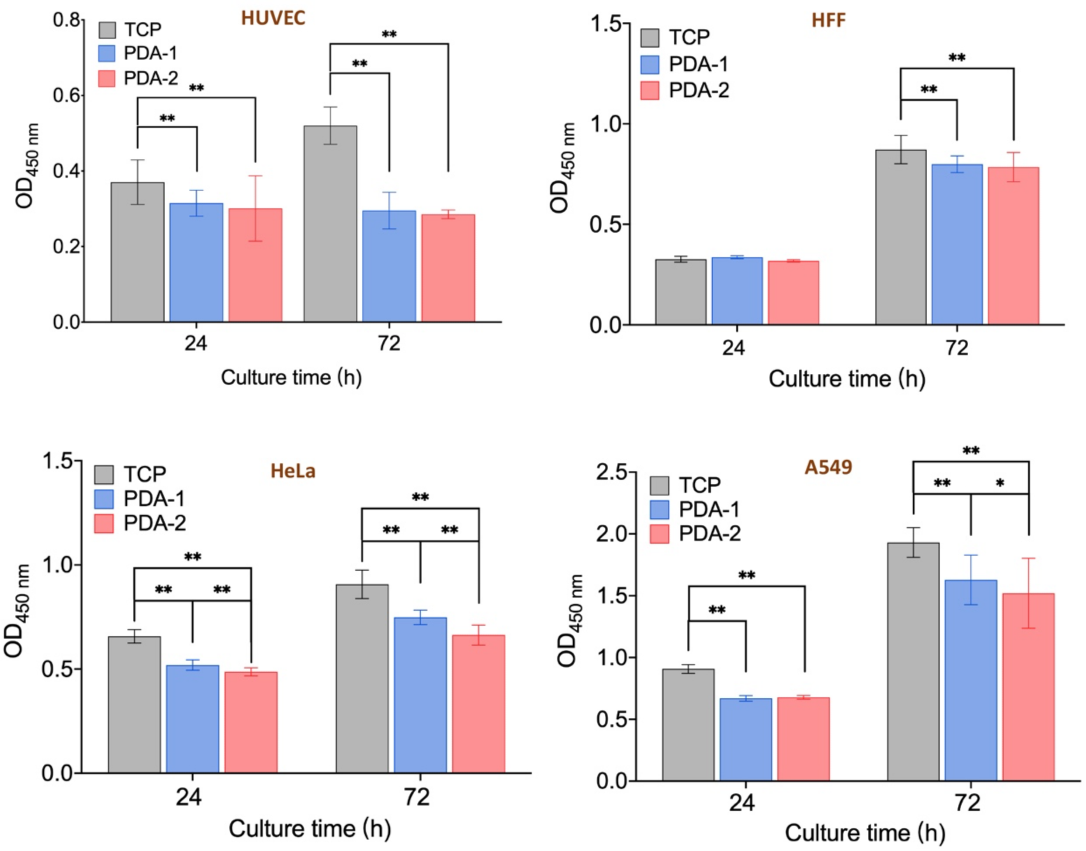

Fig. S5. Cell viabilities of the indicated different types of cell lines on PDA-modified and virgin substrates detected via CCK-8 test.

- HUVEC: human umbilical vein endothelial cells
- HFF: human foreskin fibroblast
- HeLa: a typical human cervical cancer cell line
- A549: lung cancer human alveolar basal epithelial cells

Data are presented as mean ± standard deviation and analyzed by one-way ANOVA. For each group, $n = 5$. Significant differences are marked with one asterisk ($p < 0.05$) and double asterisks ($p < 0.01$).

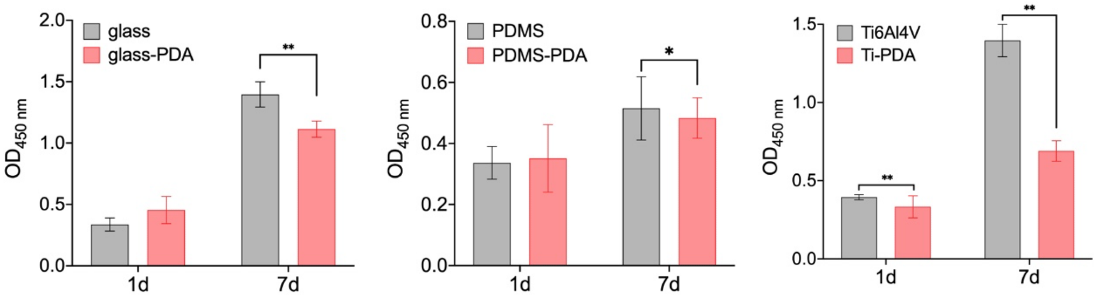

Fig. S6. Cell viability of hMSCs on the indicated other substrates detected via CCK-8 test. For each of the substrates, cells were cultured on both of the virgin surfaces and the modified surfaces. The concentration of PDA used for surface modification was 2 mg/mL.

We examined three typical biological substrates as follows:

- Glass representative of nonmetallic inorganics
- Plasma-treated polydimethylsiloxane (PDMS) representative of polymers
- Ti6Al4V representative of metals.

Data are presented as mean ± standard deviation and analyzed by one-way ANOVA. For each group, $n = 5$. Significant differences are marked with one asterisk ($p < 0.05$) and double asterisks ($p < 0.01$).

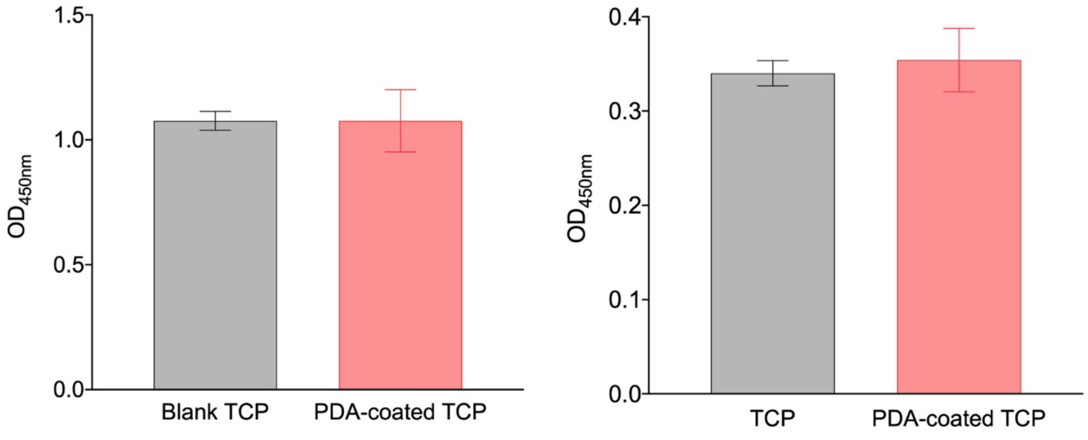

Fig. S7. Examination of possible interactions between PDA coating and the products/substrates of CCK-8 color reaction. The left figure revealed that PDA did not affect the product of CCK-8 color reaction; the right figure revealed that PDA also did not affect the substrate of CCK-8 color reaction.

Data are presented as mean ± standard deviation and analyzed by one-way ANOVA. For each group, $n = 5$. In this figure, there is no significant difference between groups.

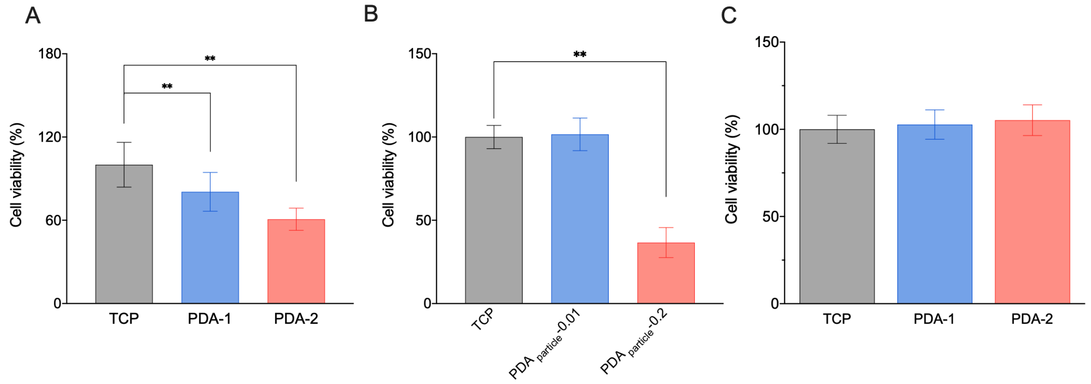

Fig. S8. Specifically designed experiments to examine the possible cytotoxicity of free PDA particles along with the PDA coating. (A) Cell viability of hMSCs cultured on repeatedly washed TCP and PDA-coated TCP substrates, which could be regarded as surfaces without free PDA particles. All the tests were carried after culturing the cells for 24 h. Data are presented as mean ± standard deviation (n = 5) and analyzed by one-way ANOVA. Significant differences were marked with one asterisk ($p < 0.05$) and double asterisks ($p < 0.01$). PDA-1 and PDA-2 mean the concentrations of dopamine in Tris-HCl were 1 mg/mL and 2 mg/mL, respectively. The 300 μL of the treatment solution was dripped on a well in a 48-well plate. Only a small fraction of dopamine was polymerized and adherent on the substrate. (B) Cell viability of hMSCs cultured with addition of pre-prepared PDA particles of the final concentrations of the particle suspensions 0.01 and 0.2 mg/mL. (C) Cell viability of hMSCs cultured with the extracts from TCP and PDA-coated (1 mg/mL and 2 mg/mL) TCP substrates. The results rule out the possibility that the decrease of cell viability on PDA-modified surfaces in other figures with respect to the PDA coating were caused from the free PDA particles. Data are presented as mean ± standard deviation and analyzed by one-way ANOVA. For each group, n = 5. Significant differences are marked with double asterisks ($p < 0.01$).

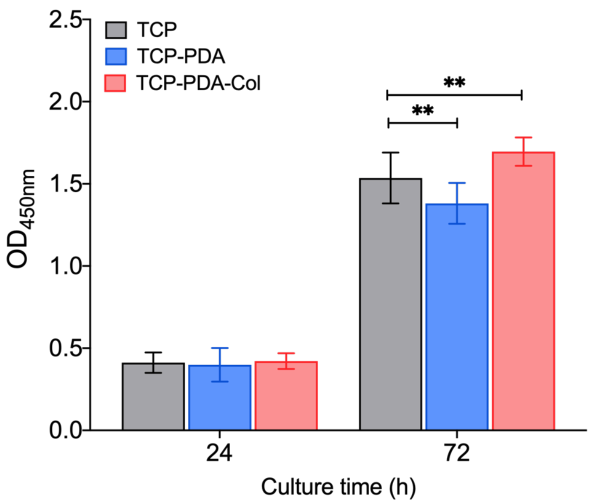

Fig. S9. Cell viability of hMSCs on TCP, PDA-modified TCP and PDA mediated collagen-modified TCP mediated by a PDA layer. The concentration of collagen was 1 mg/mL and the initial concentration of dopamine in Tris-HCl for modification was 2 mg/mL. Data detected via CCK-8 tests are presented as mean ± standard deviation and analyzed by one-way ANOVA. For each group, n = 5. Significant differences are marked with double asterisks ($p < 0.01$).

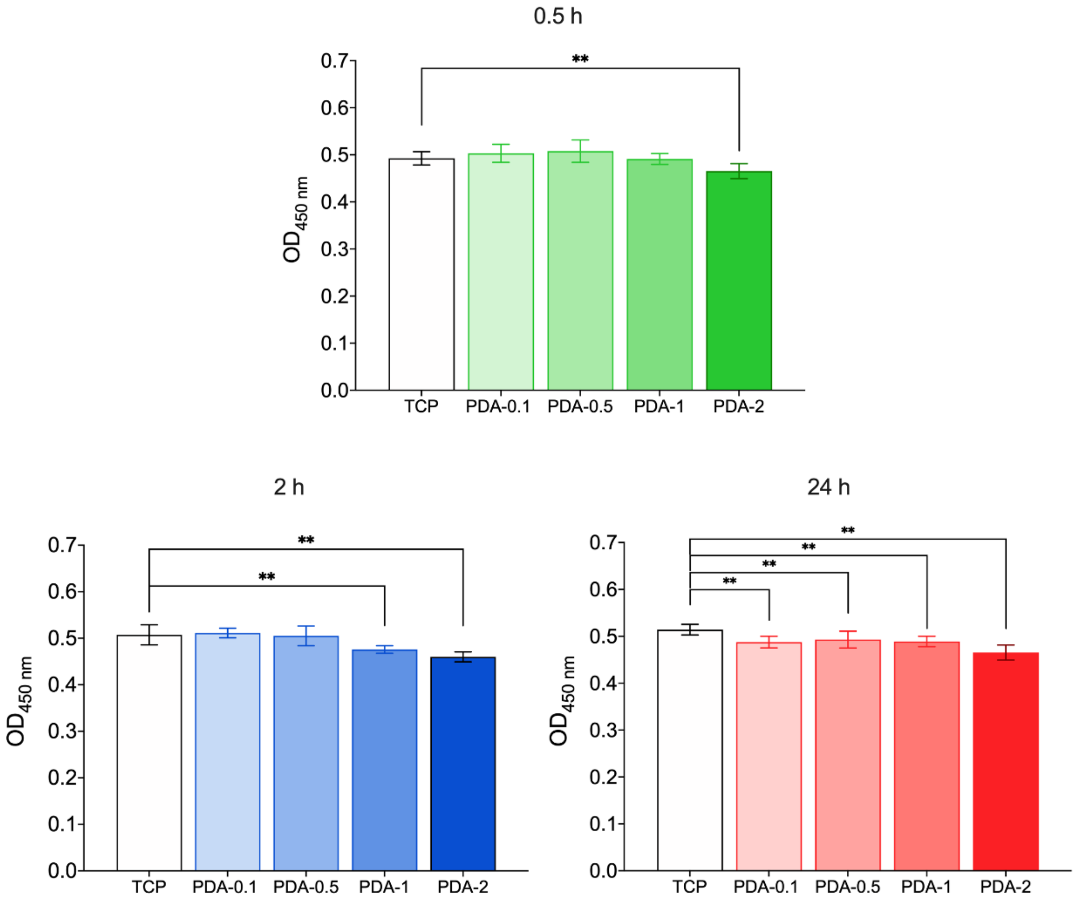

Fig. S10. Viabilities of hMSCs on TCP and PDA modified TCPs with different coating times (0.5 h, 2 h, 24 h) and dopamine concentrations (0.1 mg/mL, 0.5 mg/mL, 1 mg/mL, 2 mg/mL). Cells were cultured on these substrates for 24 h prior to the CCK-8 tests of cell viability.

Data are presented as mean ± standard deviation and analyzed by one-way ANOVA. For each group, $n = 7$. Significant differences between the virgin group (TCP) and one of the modified groups are marked with double asterisks ($p < 0.01$).

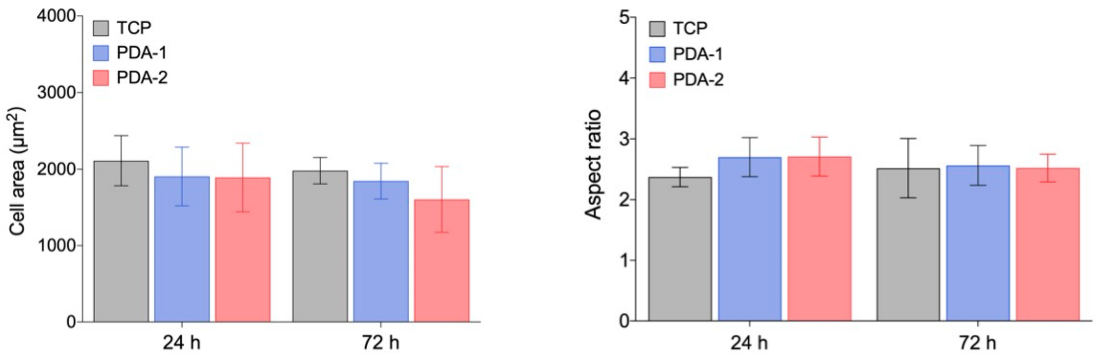

Fig. S11. Statistics of spreading areas and aspect ratios of cells on TCPs with or without PDA modification based on the fluorescence micrographs of hMSCs in **Figure 4A**. For each group, n = 5. There is no significant difference between groups.

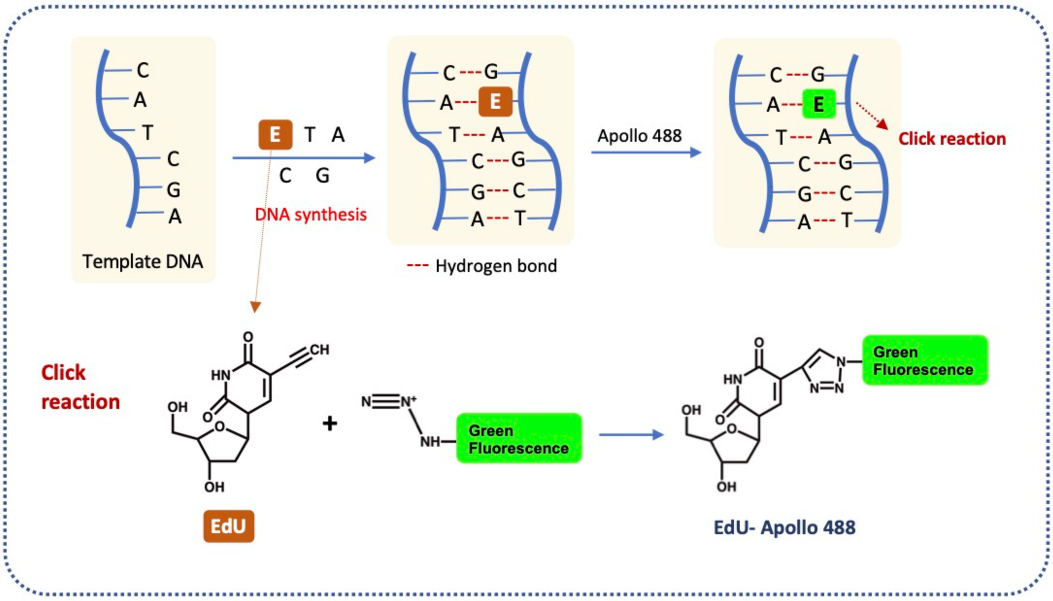

Fig. S12. Schematic presentation of the principle of EdU cell proliferation assay.

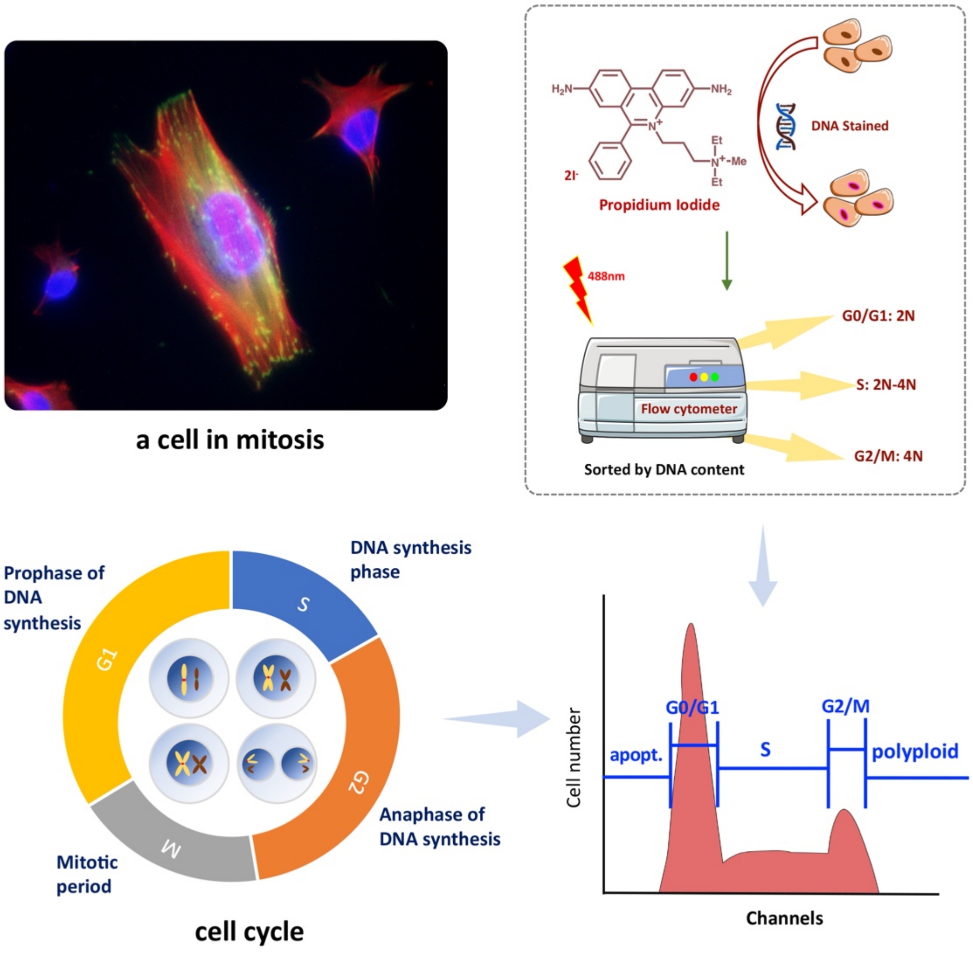

Fig. S13. Illustration of cell proliferation. The upper left shows a fluorescence micrograph of a few hMSCs on TCP, where nuclei were stained in red, F-actin in red, and vinculin in green. The central cell was experiencing mitosis, which is reflected by the double nuclei. The upper right schematically illustrates the flow cytometry detection of cell cycle, where we used propidium iodide (PI) to label DNA. The lower left indicates the four stages in a mitosis cycle, and the lower right, DNA content in cells changes periodically with the process of cell cycle. For example, the DNA content in G0/G1 phase is 2N, while that in G2/M phase is 4N. Using the PI labeling method, the relative content of DNA in cells was measured by flow cytometry, and the percentage of each phase in a cell cycle could be analyzed based on statistics of distribution in the lower right.
